# Case-Fatality Risk Estimates for COVID-19 Calculated by Using a Lag Time for Fatality

**DOI:** 10.3201/eid2606.200320

**Published:** 2020-06

**Authors:** Nick Wilson, Amanda Kvalsvig, Lucy Telfar Barnard, Michael G. Baker

**Affiliations:** University of Otago Department of Public Health, Wellington, New Zealand

**Keywords:** COVID-19, case-fatality risk, pandemic, viruses, respiratory diseases, 2019 novel coronavirus disease, severe acute respiratory syndrome coronavirus 2, coronaviruses, SARS-CoV-2, zoonoses

## Abstract

We estimated the case-fatality risk for coronavirus disease cases in China (3.5%); China, excluding Hubei Province (0.8%); 82 countries, territories, and areas (4.2%); and on a cruise ship (0.6%). Lower estimates might be closest to the true value, but a broad range of 0.25%–3.0% probably should be considered.

The coronavirus disease (COVID-19) is spreading globally; as of March 5, 2020, cases were reported in China and 85 other countries, territories, and areas (*1*). Disease severity is a particularly crucial parameter for understanding this new disease (*2*), but accurately estimating the case-fatality risk is difficult because milder cases are not being diagnosed and death is delayed.

We used data from the World Health Organization (WHO) (*1*) to calculate crude estimates of the case-fatality risk on March 5, 2020, for 4 populations: China; China, excluding Hubei Province; a group of 82 countries, territories, and areas; and passengers and crew of a cruise ship (Table). However, given the critical need to consider time lags to death when calculating case-fatality risk (*3*), we used time lags from a recent study from China (*4*). Yang et al. (*4*) reported that the median time from symptom onset to radiological confirmation of pneumonia was 5 days (interquartile range [IQR] 3–7 days); from symptom onset to intensive care unit (ICU) admission was 11 days (IQR 7–14 days); and from ICU admission to death was 7 days (IQR 3–11 days). Therefore, a median of 13 days passed from pneumonia confirmation to death ([11–5] + 7 = 13). 

**Table:** Crude and adjusted estimates of case-fatality risk for COVID-19 in 4 populations*

Location	Cumulative deaths†	Cumulative confirmed cases†	Crude CFR, %	Adjusted deaths‡	Adjusted cumulative confirmed cases‡	Adjusted CFR, % (95% CI)§
China¶	3,015	80,565	3.74	2,627	75,569	3.48 (3.35–3.61)
China, excluding Hubei Province#	113	13,099	0.86	104	12,907	0.81 (0.67–0.98)
82 countries, territories, and areas**	27	2,285	1.18	15	354	4.24 (2.58–6.87)
Cruise ship	6	706	0.85	4	634	0.63 (0.25–1.61)
*CFR, case-fatality risk; COVID-19, coronavirus disease. †Calculated by using data on laboratory-confirmed COVID-19 cases reported by the World Health Organization on March 5, 2020 (*1*). ‡Calculated by using cumulative confirmed cases as of February 21, 2020. §Calculated using OpenEpi v3 (http://www.openepi.com) by using the Score (Wilson) method. ¶Includes Hong Kong, Macau, and Taiwan. #We excluded Hubei Province because COVID-19 appears to have originated in this province and cases might have been missed because of shortages of appropriate diagnostic tests or health system overload. **Includes 82 countries, territories, and areas outside of China and reporting cases on March 5, 2020; excludes areas with >500 cases (i.e., Italy, Iran, and South Korea) because of the possibility of uncontrolled spread and missed diagnoses in these localities.						

For our calculation, we assumed that the day of radiological confirmation of pneumonia approximately equated to the reporting date for laboratory-confirmed cases of COVID-19 to WHO. We obtained cumulative COVID-19 case counts reported by WHO on February 21 (*5*), which was 13 days before March 5, the date we used for calculating the crude case-fatality risk. Our approach is broadly comparable to a study that used earlier data to estimate the median time delay of 13 days from illness onset to death (*6*).

By using the number of cumulative cases on February 21 as the denominator for the adjusted case-fatality risk (aCFR), we assumed that half of the additional cumulative reported deaths on March 5 could be matched with cases reported on February 21. We acknowledge our approach is fairly simplistic and that it can be superseded when higher quality cohort-based analyses become available.

The case-fatality risks, when adjusted for a 13-day lag time from reporting to death, were 3.5% in China; 0.8% in China, excluding Hubei Province; 4.2% in the group of 82 countries, territories, and areas; and 0.6% for the cruise ship (Table). Our result for China, excluding Hubei Province, is similar to a previous estimate of 0.9% (95% CI 0.6%–1.3%) by using a time-delay adjusted case-fatality risk for the same area (K. Mizumoto and G. Chowell, unpub. data; https://www.medrxiv.org/content/10.1101/2020.02.19.20025163v1).

Of our results, the least generalizable might be the result for China, which could be elevated because of undiagnosed mild cases, initial shortages of test kits, and elevated risk for death due to initial high demands on the healthcare system in Wuhan. The aCFR for the group of 82 countries, territories, and areas also might be affected by missed mild cases if some of the areas had undetected transmission. In terms of undiagnosed mild cases, the aCFR for the cruise ship population likely is the most accurate even though the 95% CI is broad. In addition, the aCFR for the cruise ship had a higher denominator due to inclusion of asymptomatic test-positive cases. Among 3,711 crew and passengers, 255 asymptomatic cases were identified (*7*); some of these persons subsequently might have developed symptoms. Thus, the aCFR for the cruise ship partially could reflect an infection-fatality risk. Also of note, 2,165 persons on the cruise ship were >60 years of age (*7*), and data from China indicates a much higher case-fatality risk among this age group (*8*); thus, a higher case-fatality risk might be expected in the cruise ship population than in other communities sampled. Considering these issues of generalizability, the aCFR of 0.8% for China, excluding Hubei Province, might be most accurate.

Nevertheless, given the residual uncertainties, health sector decision-makers and disease modelers probably should consider a broad range of 0.25%–3.0% for COVID-19 case-fatality risk estimates. The higher values could be more appropriate in resource poor settings where the quality of hospital and intensive care might be constrained. Higher values might also be appropriate in high-income countries with limited surge capacity in hospital services because elevated case-fatality risks could be seen at the peak of local epidemics. Because COVID-19 is expected to further spread globally, ongoing work using country-specific cohorts will be needed to more robustly clarify the case-fatality risk of this new disease.
